# Supplementary material for: Dissecting the homeodomain MAT locus and engineering novel tripolar and bipolar mating systems in Cryptococcus amylolentus
Source: mBio. 2026 Mar 17;17(4):e00059-26. doi: 10.1128/mbio.00059-26 (PMC13059729; doi:10.1128/mbio.00059-26)
Supplement: Supplemental figures — Figure S1 to S7. [file mbio.00059-26-s0001.docx]

Supplementary figures

**Figure S1. AlphaFold modeling of *C. amylolentus* Sxi1 and Sxi2 interactions.**

AlphaFold modeling of *C. amylolentus* Sxi1 and Sxi2 interactions was performed based on current annotations. Much ribbon structure is distributed at the peripheral area, the interaction interfaces, mainly between their N-terminal regions, are located at the central area (shown in this figure). AlphaFold modelled structures suggest non-self (heteroallelic) heterodimers and self (homoallelic) heterodimers of the Sxi1 and Sxi2 between and within these two isolates. There was no predicted homodimer formation between Sxi1 or Sxi2 proteins. Predicted homeobox motifs identified in the AlphaFold model are highlighted by shading.

**Figure S2.** **CRISPR-Cas9 system developed for *C. amylolentus***.

(**A)** Workflow of the Transient CRISPR-Cas9 coupled with Electroporation (TRACE) system developed for *C. amylolentus.* Briefly, the CRISPR-Cas9 system is composed of (1) the Cas9 cassette, which is expressed under the regulation of the native promoter and terminator of the *C. amylolentus TEF1* gene, (2) the sgRNA cassette containing the U6 promoter, gRNA (20 bp), scaffold and 6xT terminator; (3) the repair DNA template of 5’ and 3’ homology fused to a dominant drug marker adapted with the native promoter and terminator of the *C. amylolentus ACT1* gene. During competent cell preparation, hydroxyurea (HU, an S-phase inhibitor) and W-7 (hydrochloride, an inhibitor of the NHEJ DNA repair pathway) were included to promote homologous recombination. Transformants were validated by genotyping with specific primer sets targeting the internal region of the gene, 5’ junction and 3’ junction regions. **(B)** Deletion cassettes of individual *SXI1* or *SXI2* or both. For mutant construction in CBS6039 (left), fragments of 5’ homology, selection marker, and 3’ homology were amplified, purified and then fused by junction PCR with the indicated primer sets. For mutant construction in CBS6273 (right), fragments were fused by NEBuilder® HiFi DNA Assembly master mix, then amplified using the resulting plasmid DNA as the template. **(C)** Genotypic validation of mutant construction of *sxi1*Δ, *sxi2*Δ and *hd*Δ mutants in both CBS6039 (left) and CBS6273 (right). The correct amplification of the Open Reading Frame (ORF) of the *SXI1* or *SXI2* genes, as well as the 5’- and 3’-junction regions were confirmed using the primer sets indicated.

**Figure S3. Construction of *HD* relocated strains in CBS6039 and CBS6273.**

(**A)** Schematic diagram (left) of the DNA repair cassettes for strain generation. These cassettes were assembled into the vector pBluescript KS (-) with the NEBuilder® HiFi DNA Assembly master mix. *HD* relocation cassettes (>11 kb) were purified from plasmids using restriction enzymes XbaI and EcoRI. The *SH* deletion cassettes were amplified from a plasmid DNA template. (**B)** Genotypic validation of mutant construction of *sh*Δ and *P/R+HD* mutants in both the CBS6039 and the CBS6273 backgrounds. PCRs of *SH* region, 3’ junction and 5’ junction were conducted with primer sets as indicated.


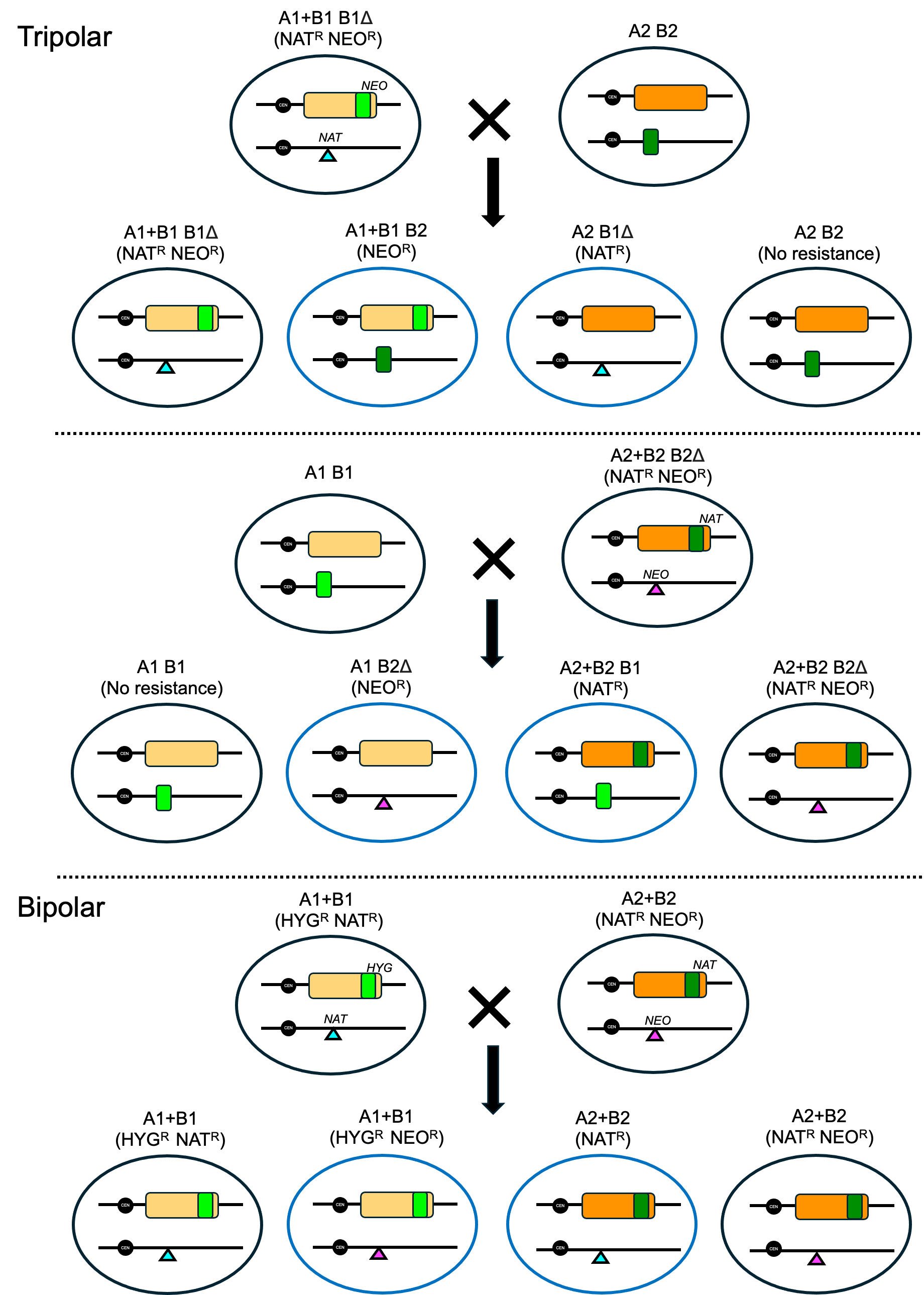


**Figure S4. Diagrams depicting *MAT* loci segregation in tripolar and bipolar crosses.**

Illustrated here are the segregations of the mating types among the progeny in the tripolar and bipolar crosses that involve engineered *C. amylolentus* isolates containing modified *MAT*. The yellow and orange rectangles indicate the A1 and A2 *P/R* wild-type alleles, while the light green and dark green rectangles indicate the B1 and B2 *HD* wild-type alleles, respectively. For genetically modified alleles, the light blue and purple triangles indicate the deletion of endogenous B1 and B2 *HD* alleles with the *NAT* and *NEO* markers, respectively; the light green bar within the A1 *P/R* allele indicates the insertions of the B1 *HD* genes into the A1 *P/R* locus (using the *NEO* or *HYG* marker), and the dark green bar within the A2 *P/R* allele indicates the insertion of the B2 *HD* genes into the A2 *P/R* locus (using the *NAT* marker).

**Figure S5. Phenotypic analysis of F1 progeny harboring two sets of *HD* genes.**

**(A)** Solo culture of 15 F1 progeny inherited the “A2+B2 B1” mating type growing on V8 (pH 5) medium and incubated in the dark at room temperature for 2 weeks. Scale bar: 100 µm. **(B)** Solo culture of 20 F1 progeny inherited the “A1+B1 B2” growing on V8 (pH 5) medium and incubated in the dark at room temperature for 2 weeks. Scale bar: 100 µm.


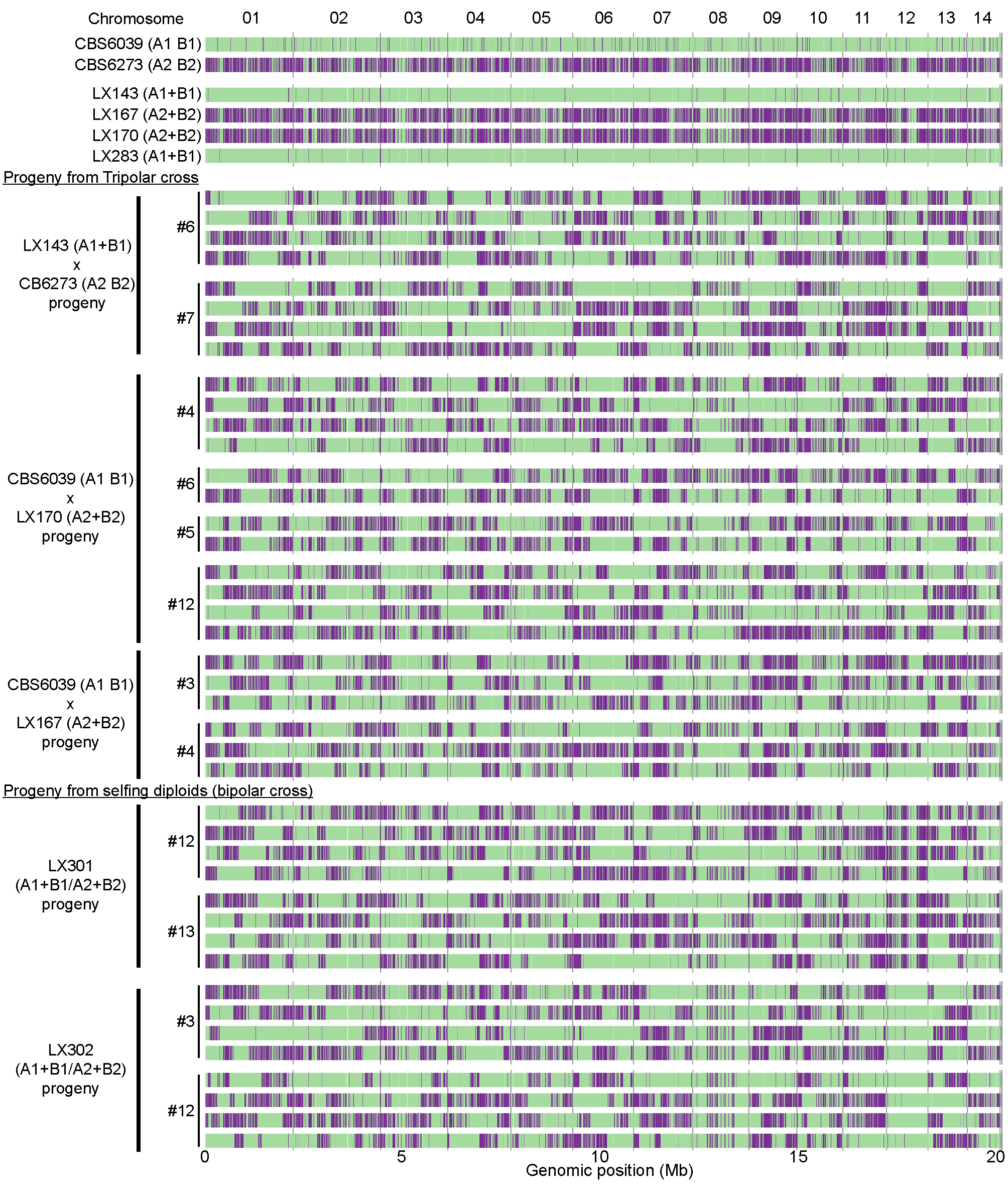


**Figure S6. Variant mapping illustrates genome-wide meiotic recombination in progeny from tripolar and bipolar crosses in *C. amylolentus*.**

SNP patterns of progeny from tripolar and bipolar crosses reveal crossovers consistent with meiotic recombination. Green and purple correspond to variants from CBS6039 and CBS6273, respectively. Parental strains of different mating types and their respective progeny are shown on the left. The numbers of basidia sampled are indicated (#) and correspond to those listed in Tables 1 and 2. For tripolar crosses, both representative basidia and random basidiospores were analyzed; for selfing diploids (A1+B1/A2+B2), eight progeny from two basidia each were examined. *P/R* locus and centromeric (*CEN*) regions are highlighted in shade.


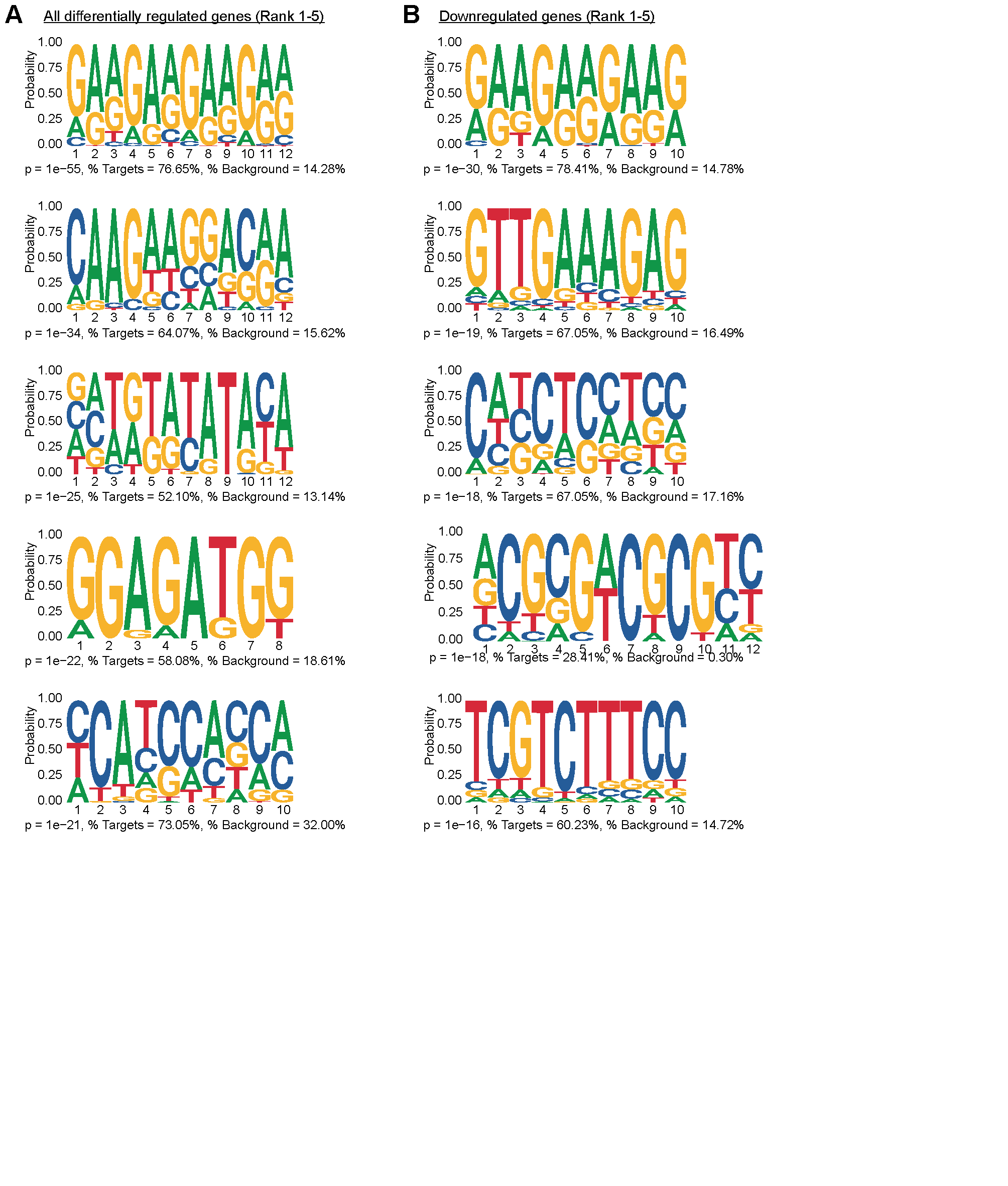


**Figure S7. Predicted binding motifs of Sxi1-Sxi2 responsive genes under mating-inducing conditions.**

Motif enrichment analysis was conducted using MEME on the promoter regions (1 kb upstream of the start codon) of all differentially expressed genes **(A)** and down-regulated genes **(B)** in *hd*Δ mutant under mating inducing conditions. The top 5 enriched sequence motifs (rank 1-5) are shown as sequence logos, representing potential binding sites associated with Sxi1–Sxi2–dependent gene regulation. Motifs are ranked by statistical significance (p value), and the coverage of targets (%) and background (%) are indicated below each motif logo.
